# Social and Poor Law Problems

**Published:** 1906-08-25

**Authors:** 


### SOCIAL AND POOR LAW PROBLEMS.

### "EVERY COMFORT" FOR THE PAUPER CHILD.

One of the witnesses before the Local Government Board Inspector who is inquiring into the affairs of Poplar, a lady, was asked if she thought that "a girl dining in a hall with carvings equal to an Oxford college, and having tiled baths, was being fitted for domestic service." She replied with the amiable generalisation that "she thought pauper children should have every comfort." We do not know what this lady included in "every comfort," but evidently the two items mentioned by the Inspector were not outside it. The trifling fact that pauper children enjoyed luxuries that were not possessed by the children of those who supported them gave her no qualms. Yet, as a matter of simple justice, it is not fair that the recipient of charity should fare better than the donor. But the point on which the Inspector touched is a far more serious one. Do the luxuries which pauper children enjoy during their school-days fit them or unfit them for their future conditions of life. "Every comfort" has more than a temporary and physical effect; it has a permanent moral one as well. If a girl finds, when she goes out to earn her living, that she is less well off than when she was maintained by the parish, she cannot be blamed for wishing to return to dependance again. It is merely human to desire the comforts we have known. The boys and girls of self-supporting classes who find service, or the shop, or the office, less comfortable than the home they have left, know also that if they are ever to enjoy these comforts again they must earn them for themselves. If they even envy the comforts possessed by their employers, they may be incited to greater energy in the hope of obtaining an equal reward. But the comforts of pauperism contain no such lesson. On the contrary, the inference is that the death or unworthiness of parents is rather an advantage, that the Guardians are their protectors against the world, and that when things do not go comfortably, the best thing they can do is to appeal again to the Guardians for help. Such kindness is a hidden cruelty. It must not be supposed that Poplar is the only Union where the children are treated indulgently. Let two instances from another Union exemplify this. A lady Guardian, visiting a school where some children from her parish were boarded, turned up the beds to see merely if they were clean and decent. The matron of the school, who was present, explained in a tone of anxious apology that these beds were not as good as some. "We have spring mattresses in most of the dormitories, but these beds were quite good when we got in the springs, and it didn't seem quite right, did it? to throw them away. But we are replacing them as fast as they are worn out." The Guardian thought of the attic room and the hard bed which would probably be the lot of the occupant of that bed before many months had passed, and thought that no apology was necessary. Again, a boy, apprenticed to a respectable master, wished to have the apprenticeship broken. He made many complaints, but the most definite fault was that "he did not get his bath comfortably." He had himself to carry the water into a portable bath in the scullery, and he had been used, at school, to a proper bath-room, with hot and cold water on tap. Therefore he could not endure treatment which, if he had come straight from his home, would have seemed to him quite good enough. This is the moral effect of "every comfort."

### MIDWIVES *VERSUS* SANITARY INSPECTORS.

It would not occur to the average mind that there was either competition or hostility between the midwife and the lady sanitary inspector, yet at a meeting of the Bootle Town Council one of the members, Dr. Rafter—can he be a medical man?—objected to the appointment of an additional female sanitary inspector, as recommended by the Health Committee, and recommended that midwives should be appointed instead of sanitary inspectors. Most people will agree with Dr. Rafter in wishing that there were more trained midwives available for the poor, but few will agree that an addition to the ranks of these should be purchased at the cost of sacrificing lady sanitary inspectors. Dr. Rafter seemed to regard the sanitary inspector as merely an interfering person "rigged out and made to look as imposing as possible," to use his own words, who intrudes into the homes of the poor. Even if she were not more than a health visitor, a female inspector would do good work, if, through pride, or the irritation of being reproved, her visits made people keep their houses cleaner than they otherwise would; but, as a matter of fact, the sanitary inspector does more than this, and a woman can often dive into matters which would not occur to a man. Anything that makes for cleanliness should surely be supported by the medical profession, as helping on the cause of public health. Both as sanitary inspectors and factory inspectors, women have done, and are doing, admirable work, and work which seems to have been left undone until they were appointed, or why should it be there to do? Some recent reports of women inspectors as to the condition of labour in sweet and jam factories show that there is much in these places for a woman's eye to note and a woman's voice to condemn. One lady says:—"Festoons of black cobwebs of large size in rooms where sweets are made and stored, as well as in passages and lavatories, show how absolutely the most elementary cleansing is neglected. As do also the floors heaped with dirt and refuse. I shamed the manager into giving orders for an immediate sweeping and cleansing of floors and walls." Another inspector discovered that the water in which dirty jam-pots were washed was changed "about once a week." This water "smelt abominably," yet the pots, when taken out of the water, were simply allowed to dry before being refilled with jam. It is just such details as cobwebs and dirty dish-water that women inspectors take more notice of than men, and while they continue to do so, either in the factory or the home, we may well protest against any attempt to limit their number.

